# Assessing Immunological Memory in the Solitary Ascidian *Ciona robusta*

**DOI:** 10.3389/fimmu.2019.01977

**Published:** 2019-08-16

**Authors:** Daniela Melillo, Rita Marino, Giacomo Della Camera, Paola Italiani, Diana Boraschi

**Affiliations:** ^1^National Research Council (CNR), Institute of Biochemistry and Cell Biology (IBBC), Naples, Italy; ^2^Biology and Evolution of Marine Organisms (BEOM), Stazione Zoologica Anton Dohrn, Naples, Italy

**Keywords:** *Ciona robusta*, innate immunity, immunological memory, innate memory, immune priming

## Abstract

The immune defensive mechanisms active in the solitary ascidian *Ciona robusta* include phagocytic and encapsulating activity, largely brought about by phagocytic cells within the haemocyte population, the presence of complement components, which have been molecularly and functionally identified, and expression of a number of immune-related genes and pathways, identified by genome-based homology with vertebrate counterparts. Since *C. robusta* only displays highly conserved innate immune mechanisms, being devoid of an adaptive immune system, this organism is an excellent model for studying the features of innate memory, i.e., the capacity of the innate immune system to re-programming its responsiveness to potentially dangerous agents upon repeated exposure. In this study, we have developed an *in vivo* model for assessing the establishment and molecular/functional features of innate memory, by sequentially exposing *C. robusta* to a priming stimulus (microbial molecules), followed by a period of resting to return to basal conditions, and a challenge with microbial agents in homologous or cross-stimulation. The endpoints of immune activation were a functional activity (phagocytosis) and the molecular profiles of immune-related gene expression. The results show that exposure of *C. robusta* to microbial agents induces a reaction that primes animals for developing a different (expectedly more protective) response to subsequent challenges, showing the effective establishment of an immune memory. This immune memory relies on the modulation of a number of different mechanisms, some of which are priming-specific, others that are challenge-specific, and others that are non-specific, i.e., are common to all priming/challenge combinations (e.g., up-regulation of the *Tnf* and *Lbp* genes). Memory-dependent expression of the humoral immunity-related gene *C3ar* inversely correlates with memory-dependent variations of phagocytic rate, suggesting that complement activation and phagocytosis are alternative defensive mechanisms in *C. robusta*. Conversely, memory-dependent expression of the cellular immunity-related gene *Cd36* directly correlates with variations of phagocytic rate, suggesting a direct involvement of this gene in the functional regulation of phagocytosis.

## Introduction

The metazoan immune defence system reaches its highest level of complexity in vertebrates, which exploit the integrated activity of innate and adaptive immunity. Conversely, invertebrates rely on the innate immune system as the sole immune defence mechanism (1–4). The innate immune system provides a first line of rapid and non-specific defence (5), while the adaptive immune system is slower, antigen-specific, and endowed with immunological memory that confers long-term protection. Against the dogma that innate immunity has no memory, an increasing number of studies have confirmed the existence of an innate immune memory both in vertebrates (6–10) and in several invertebrate species (11–14). Indeed, the fact that over 99% of living diversity only relies on innate immunity supports the concept of innate memory (14).

The defensive inflammatory response triggered by exposure to a stimulus might be enhanced (“potentiation,” “trained immunity”) or decreased (“tolerance”) upon exposure to subsequent challenges, showing the establishment of an immune memory that aims at improving protective reaction to potential dangers (15). The study of the immune memory in invertebrates is of particular interest since these species lack adaptive immunity and adaptive immune memory, thus innate immune memory is their only form of immunological memory. This allows us to best examine its biological and molecular mechanisms (some of which are well-conserved among the different species).

In this context, we have focused on a marine invertebrate, the ascidian *Ciona robusta* [previously known as *Ciona intestinalis*; (16, 17)] to assess the features of establishment and activity of immunological memory. This tunicate tolerates a broad range of both temperature and salinity, has a fast growth rate and high fecundity and, more interesting from an immunological point of view, it lives well in polluted environments. In fact, being a sessile filter-feeding animal in its adult stage, *C. robusta* is continuously exposed to microbial loads (18). Hence, *C. robusta* represents a good model for assessing the establishment of protective immune memory in response to multiple microbial challenges.

Ascidians are invertebrate chordates phylogenetically close to vertebrates, with a larval stage possessing the basic developmental and morphologic features of vertebrates, a fact that has fostered extensive studies by developmental and evolutionary biologists (19). Consequently, its whole genome (160 Mbp, 14 pairs of chromosomes, about 16,000 genes) is well-annotated. Thus, bioinformatic approaches and extensive *in silico* search have allowed for identifying a number of immune-related molecules and gene expression patterns. Notably, only in few cases the immune molecules were identified and characterised functionally (20–24).

The external surface of ascidians is the tunic, a special mantle composed of cellulose, proteins, and inorganic compounds, which is an effective mechanical barrier against environmental microorganisms (25, 26). Tunic injury and/or injection of foreign substances across the tunic barrier cause fluid and blood cells (haemocytes) accumulation at the injury site (27–30). This inflammatory response is a key evolutionary innate immunity model that allows us to study the mechanisms of clearance of foreign substances by haemocytes (by phagocytosis or encapsulation) and the role of their putative paracrine and autocrine products (31–34). Since no pathogenic microorganisms have been identified in *C. robusta*, studies have been conducted with prototypical microbial agents. Lipopolysaccharide (LPS), a component of the cell wall of gram-negative bacteria, could stimulate the release of the defensive enzyme phenoloxidase by ascidian haemocytes *in vitro* (35). *In vivo*, LPS induces inflammatory reactions with the up-regulation of immune-related genes (24, 36, 37).

In vertebrates, LPS and other microbial molecules (collectively defined as PAMP, pathogen-derived molecular patterns) bind to Toll-like receptors (TLRs), as well as other innate receptors, on immune cells. Toll (discovered in *Drosophila*) and Toll-like receptors are abundantly present in invertebrates. In *C. robusta*, three Toll-like genes have been recognised. *Tlr-1* and *Tlr-2* have been cloned (38). Although sharing the characteristics of TLR genes, they have no clear homology to any of the vertebrate TLR genes (39). From *in silico* structural characterisation and studies on human cells transfected with *Ciona* TLRs, it appears that these two TLRs are present both on the plasma membrane and a number of late endosomes, can recognise a more extensive PAMP range than vertebrate TLRs, and trigger NF-κB transactivation upon PAMP binding (38, 39). The third TLR gene, *Tlr13*, has been predicted by sequence homology with the murine *tlr13* gene, but it has not been cloned nor functionally characterised (40).

Among the major defensive effector mechanisms of innate immunity, several complement genes were identified in the ascidians, belonging to both the alternative and lectin complement activation pathways (13, 20, 22, 41–44) and few of them were also identified as proteins and functionally characterised (20–24). The gene encoding C3-1 is expressed in haemocytes, and also in several organs, including pharynx (45). Immunohistochemical analysis revealed that the complement anaphylatoxin C3-1a and its receptors are significantly expressed in haemocytes and functionally involved in chemotaxis (21). Other putative immune-related genes were identified in the *C. robusta* genome, and some of them cloned. These include the anti-inflammatory factor transforming growth factor β (*Tgfb*) (46), and the inflammatory cytokines interleukin 17 (37) with three homologous genes (*Il17-1, Il17-2, Il17-3*), tumour necrosis factor α (*Tnf*) (23) and the receptors for IL-17 (*Il17r*) (47). *Tgfb* is transcriptionally up-regulated during inflammation induced by an LPS inoculum, suggesting that it is involved in the inflammatory response (46). The expression pattern of these immune-related genes in organs from adult individuals has allowed us to identify additional events activated during inflammation. Besides the influx of some circulating haemocytes (undifferentiated cells, amoebocytes and vacuolated cells) to the area of injury, haematogenic sites (crypts or nodules and haematopoietic cells clusters in the pharynx) are also directly involved in inflammatory reactions (24). Indeed, the formation of haematopoietic nodules in the lumen of *Tnf*-expressing vessels suggests a possible involvement of TNFα in promoting haemocyte differentiation (24).

In this study, we approached the analysis of immunological memory in *C. robusta* by repeated exposure to prototypical microbial stimuli both in homologous and heterologous combination. We evaluated the establishment and effects of immune memory by functional characterisation (haemocyte composition and phagocytosis) and expression profiling of 10 immune-related genes. These include two genes encoding proteins known to participate in immune responses, based on functional studies (*C3-1, C3ar*), some genes whose expression is increased upon *in vivo* stimulation (*Il17, Tnf*, *Tgfb*), and *Tlr-2*, whose involvement in an immune response (NF-κB transactivation) has been assessed *in vitro* in HEK-293 cells transfected with the ascidian gene (38). In addition, we have examined four genes that, from sequence homology, are predicted as being immune-related, namely *Il17r, Tlr13, CD36*, and *Lbp* (accession numbers are listed in Table 1). From our data we conclude that priming induces a variation of both cellular and humoral defence responsiveness to subsequent challenges, likely aiming at better coping with potential dangers. This appears to be a shift towards more phagocytosis and enhanced cellular immune responses against gram-negative challenges, and a shift towards decreased phagocytosis and increased complement and humoral immunity in response to gram-positive agents.

**Table 1:** List of primers used for evaluating gene expression in *C. robusta*.

**Gene**	**Forward**	**Reverse**	**Genbank acc. No**.
*C3-1*	5′ -acagacgtggcgtgtgcaag-3′	5′-tactttgcctaggaggccggt-3′	AJ320542
*C3ar*	5′ -ttgccccgccatgcgagga-3′	5′-aggtacgactccatacaacacc-3′	AJ966353
*Il17-2*	5′-cgggtgcattgcttctagt-3′	5′-cacgcaggtacagcctattg-3′	NM_001129874.1
*Il17r*	5′-gtgacccgtggcaatcaatgg-3′	5′-caagttaggcattttgctccgt-3′	AY261862
*Tnf*	5′-catctccccaccctactacac-3′	5′-atttgcgcaaacgtctggca-3′	AM982527
*Tgfb*	5′-ctcgttcaaatgtgtctcaaaccg-3′	5′-cgttgccagattttacgacg−3′	AB210727
*Lbp*	5′-ggtttcgggaagctgggatt-3′	5′-gaaggggcctgtttcttcca-3′	XM_002126995.2
*Tlr-2*	5′-acgcaagaaacaagagagacg-3′	5′-gcttttcttccatttcctccagc-3′	AB495262.1
*Tlr13*	5′-cggaagcattgtgctggaaa-3′	5′-acgcaagacaaatacgcctg-3′	XM_002120484.4
*Cd36*	5′-ggttcgcttttatttcttggacct-3′	5′-ctgcaccgtttggtttacgg-3′	XM_009860510.1
*Gapdh*	5′-cattttcgacgcaggagctg-3′	5′-ctgcgtggtgtttaactggc-3′	XM_002131188.4

## Materials and Methods

### Animals and Treatments

Adults of *C. robusta* were collected in the small sea of Taranto, and maintained in the Zoological Station of Naples in circulating seawater at 18°C. Haemolymph samples (100 μl) were collected with a syringe from the perivisceral cavity of 10 animals for each experimental group before starting the treatments. The actual concentration of haemocytes in haemolymph of control and treated animals was exceedingly variable and independent of the treatment, most likely depending on the sampling procedure, and therefore is not reported.

To induce an inflammatory reaction (priming step), animals were administered with 25 μg of lipopolysaccharide (LPS, from *Escherichia coli*, serotype O55:B5; Sigma Aldrich Inc., St. Louis, MO, USA), 25 μg of lipotheicoic acid (LTA, from *Bacillus subtilis*; Sigma Aldrich), 50 μg of the fungal particulate β-glucan Zymosan (from *Saccharomyces cerevisiae*; InvivoGen, San Diego, CA, USA) or 50 μg of the synthetic virus-like dsRNA Poly(I:C) (HMW; InvivoGen). Stimuli were inoculated in 50 μl of marine solution (MS: 0.45 M NaCl, 26 mM MgCl_2_, 11 mM KCl, 12 mM CaCl_2_, pH 7.4), through the tunic between the two siphons. The dose of stimuli was selected from preliminary dose-response experiments as the highest non-lethal dose that provoked a visible reaction in the animals (a transient collapse, with recovery in few hours). Control individuals were injected with the same volume of MS. Haemolymph samples were collected again 24 h after treatment. Animals were kept in tanks containing aerated seawater for additional 7 days (resting or extinction period). This period was selected from preliminary experiments as sufficient for returning of stimulus-induced functional and molecular responses to background level. Before challenge, haemolymph was collected again and the haemocyte phagocytic capacity tested as described below, to confirm the return to basal conditions. Animals were then challenged with a double dose of LPS or LTA (50 μg in 50 μl). Animals primed with the non-bacteria stimuli Zymosan and Poly(I:C) were only challenged with 50 μg of LPS. The challenge dose of either stimulus resulted in about 90% survival in non-primed animals. For gene expression analysis, collection of pharynx samples required the animals' sacrifice, and therefore replicate groups were set up.

### Bacterial Cultures and Labelling

*Escherichia coli K12* and *Bacillus cereus* bacteria were grown overnight in Luria-Bertani medium at 37°C in a rotating shaker incubator. After heat-inactivation at 60°C for 1 h, bacteria were washed twice by centrifugation at 3,000 rpm for 10 min at 4°C and resuspended in MS. For labelling, bacterial suspensions (1 × 10^9^ bacteria in 1 ml MS) were incubated with 2.5 μl of 10 mM fluorescein isothiocyanate (FITC; Sigma Aldrich) in dimethylsulfoxide for 30 min at room temperature (RT) in the dark. Bacteria were then washed as described above, and resuspended in MS at the concentration of 5 × 10^7^/ml. Successful labelling was checked with an epifluorescent microscope.

### Phagocytosis

The *in vitro* phagocytosis assay was performed as follows. Haemolymph from 10 animals for each treatment was immediately diluted 1:1 in MS. Haemocyte composition (percentage of phagocytes, and percentage of granular and hyaline phagocytes) was evaluated with a light microscope at 40 × magnification, and their total number assessed by counting with a Neubauer chamber. Cells were diluted to a final concentration of 1.5 × 10^6^ cells/ml, and 100 μl dispensed in silicone isolators (Sigma Aldrich) previously fixed on super-frost slides (Thermo Fisher Scientific, Waltham, MA, USA). After adherence for 15 min at 18°C in a humid chamber, slides were washed with MS, and FITC-labelled *E. coli* K12 or *B. cereus* bacteria (5 × 10^6^ bacteria/100 μl MS) were added and incubated for 1 h at 18°C in a humid chamber in the dark. Slides were then extensively washed with MS to eliminate free bacteria, and fixed with 3.7% formaldehyde in MS for 20 min at RT in the dark. After detaching the silicone isolators from slides, cells were washed with MS, and covered with one drop of mounting medium with DAPI (Vector Laboratories, Burlingame, CA, USA). Finally, slides were mounted with a coverslip and stored at 4°C until observation.

All experiments were performed in duplicate for each treatment. Phagocytosis was quantified by counting the number of phagocytosing and non-phagocytosing haemocytes in 10 random fields with an epifluorescent microscope at 40 × magnification. Phagocytic rate (PR) was calculated as percentage of phagocytosing cells within the total phagocyte population, whereas the phagocytic index (PI) was calculated as the number of phagocytosed bacteria per phagocytosing cell.

### RNA Extraction and cDNA Synthesis From Pharynx

Three animals from each treatment at every time point were sacrificed, and fragments of the pharynx were collected (about 0.5 cm^3^ from the areas around the injection site). Samples were weighed and immediately homogenised with an Ultra-Turrax T25 at 0°C with 3 cycles of 30 s, then processed for total RNA extraction with commercially available kits (miRNeasy Kit; Qiagen, Hilden, Germany), according to the manufacturer's instructions. A mix of Oligo (dT) and random-primed single-stranded cDNA were synthesized from 2 μg of pharynx RNA using the QuantiTect Reverse Trascription Kit (Qiagen).

### Real-Time PCR

Real-time PCR experiments were carried out with a RotorGene instrument (Qiagen) with RealAmp qPCR Master mix chemistry (GeneAll Biotechnology Co., Ltd., Seoul, South Korea). Specific primers were designed, according to the nucleotide sequence, for genes encoding the *C. robusta* homologues of C3-1 (*C3-1*), C3aR (*C3ar*), IL-17 (*Il17-2*, the second isoform found in *C. robusta*), IL-17R (*Il17r*), TNFα (*Tnf*), TGFβ (*Tgfb*), LBP (*Lbp*), TLR-2 (*Tlr-2*), TLR13 (*Tlr13*), and CD36 (*Cd36*) (Table 1). Gene nomenclature is designed according to previous publications (21, 37, 38, 42, 46–48) and includes the indication of the *C. robusta* gene isoforms as a number after a dash. Other isoforms of C3 (*C3-2*), IL-17 (*Il17-1, Il17-3*), and TLR (*Tlr-1*) could not be evaluated because their expression resulted undetectable in every condition in both haemocytes and pharynx. Likewise, expression of the gene encoding the precursor of the enzyme phenoloxidase was not detectable. After preliminary evaluation of different housekeeping genes (*Actin, S27, Gapdh*), the glyceraldehyde 3-phosphate dehydrogenase gene *Gapdh* was selected for its consistent expression stability, and used as reference gene in all experiments. All primers produced single-band amplicons of the expected size and were verified by DNA sequencing. Reactions were performed in triplicate, and the PCR programme included a denaturation step (95°C for 15 min) followed by 40 cycles of amplification (94°C for 15 s, 60°C for 30 s, and 72°C for 30 s), and a final extension step (72°C for 10 min). PCR amplification efficiencies, calculated for primer pairs of the reference and target genes, were both 2. All data were normalised against *Gapdh* using the Pfaffl method. Real-time PCR results are reported as relative gene expression towards *Gapdh* (Supplementary Table 1), and ratio between treated and control animals (**Figures 5**–**7**).

### Statistical Analysis

All values were expressed as mean ± SD of samples from 3 to 20 individual animals. Statistical significance of differences between treatments was assessed by using the Student's *t* test followed by non-parametric Mann-Whitney *U*-test for phagocytosis rate and index data, and one sample *t* test for PCR data. Statistics was analysed using the GraphPad Prism 6 software. *P* values are reported in the figures, as recently recommended (49).

## Results

### Setting an *in vivo* Experimental Model of Innate Memory

**Figure 1:**
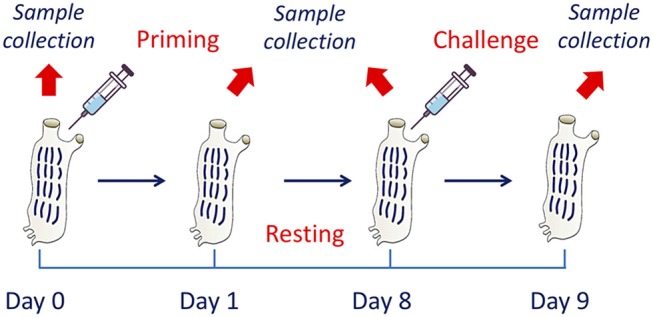
The *in vivo* model of innate memory in *C. robusta*. An *in vivo* model of establishment of innate memory was designed and optimised. This includes treatment of animals at time zero (priming), a period of resting (until day 8) to allow extinction of the response, and a second treatment (challenge). Biological samples were collected at the beginning of the experiment (before priming) for the evaluation of the basal conditions, at day 1 for evaluating the activation due to priming, at the end of the resting period to evaluate the return to basal conditions, and 1 day after challenge to evaluate the changes in the response due to previous priming.

To assess the development of innate memory, animals were sequentially exposed to inflammatory stimuli *in vivo*. After a number of preliminary dose-response and time-course experiments (not shown), the following model was optimised and adopted (Figure 1). At time zero, after collection of a sample of haemolymph, animals were primed with a cross-tunical injection of prototypical microbial stimuli in MS (controls receiving MS alone), and activation examined after 24 h (phagocytic activity of haemocytes, gene expression in pharynx). Primed animals were then rested for 7 days to allow return to baseline conditions (extinction phase). Haemolymph and pharynx samples were collected again after resting (to check response extinction), and animals were then challenged cross-tunically with 50 μg of LPS or LTA (double as compared to the primary challenge). Memory-dependent activation was tested after 24 h (phagocytosis, gene expression). Ten animals were included in each experimental group. While for the phagocytosis experiments the same animals were tested throughout (as haemolymph collection did not imply sacrificing the animals), replicate groups were necessary for evaluating gene expression, as pharynx collection required animal sacrifice.

### Innate Memory Affects Haemocyte Composition and Phagocytosis

**Figure 2:**
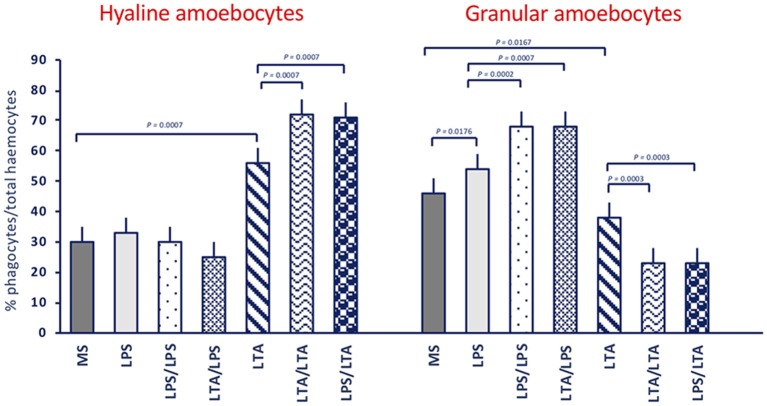
Phagocyte populations in haemocytes of *C. robusta* exposed *in vivo* to LPS or LTA. Changes in the percentage of the two different phagocyte populations within haemocytes in response to *in vivo* exposure of *C. robusta* to marine solution (MS, control), LPS or LTA alone and in different priming/challenge combinations. **Left panel**: changes in the percentage of hyaline amoebocytes. **Right panel**: changes in the percentage of granular amoebocytes. Data are the mean ± SD of five animals.

In control animals (receiving MS), the majority of adherent haemocytes (76%) was represented by the two types of phagocytic cells, the hyaline (30%) and the granular amoebocytes (46%) (Figure 2). In unprimed or MS-primed animals challenged with LPS or LTA, the overall percentage of phagocytic cells increased to about 87–94%. In response to LTA, an increase in hyaline cells is evident (from 30 to 56%) with a corresponding decrease in granular amoebocytes (from 46 to 38%), while in response to LPS only an increase in granular cells could be seen (from 46 to 54%). In animals primed with either LPS or LTA, challenge with LPS did not change the percentage of hyaline cells (25–30%) but significantly increased the percentage of granular amoebocytes (68%). Conversely, primed animals responded to LTA with a significant increase in hyaline cells (71–72%) and a concomitant decrease of granular cells (23%). Images of amoebocytes from control and LPS-treated animals that have phagocytosed *B. cereus* are presented in the Supplementary Figure 1. As the two cell types are morphologically distinguishable, it is evident that granular cells are those that actively phagocytose bacteria, whereas phagocytosis by hyaline cells is limited.

**Figure 3:**
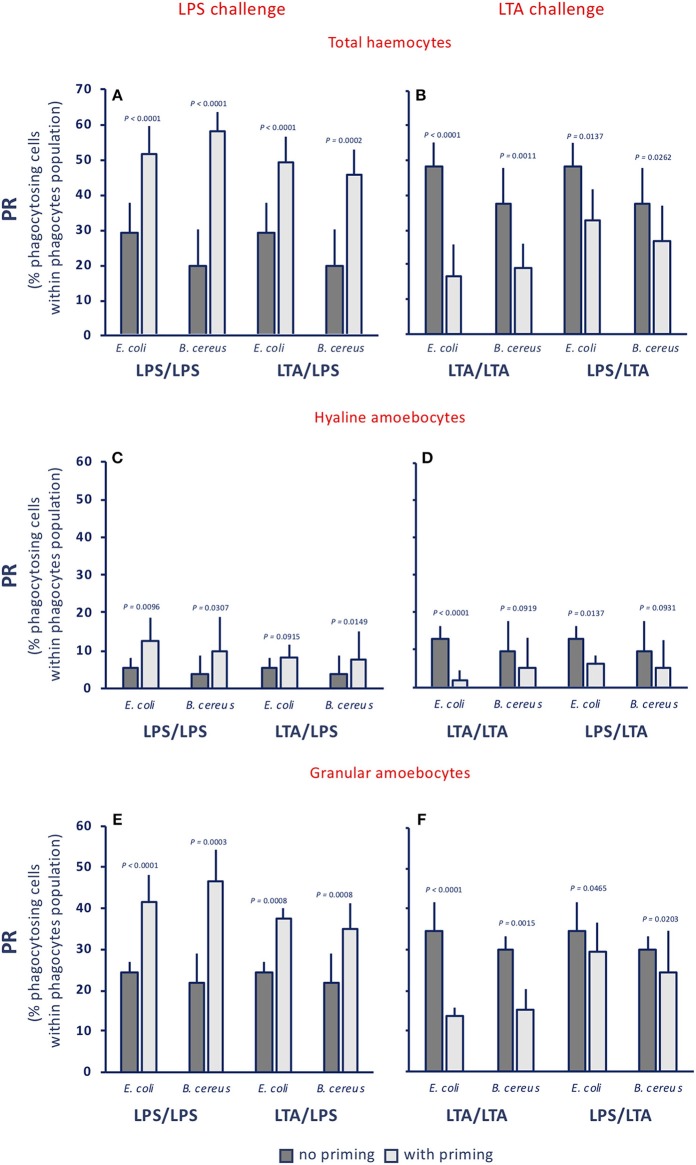
Innate memory-induced variations in the phagocytic rate of haemocytes of *C. robusta* exposed *in vivo* to LPS or LTA. The phagocytic rate (PR), i.e., the percentage of phagocytosing cells within the phagocyte population in haemolymph, was assessed 24 h after primary exposure to LPS or LTA (dark grey columns, no priming), and 24 h after homologous or heterologous challenge of primed animals (light grey columns, with priming). Phagocytosis of two bacterial strains was evaluated, the gram-negative *E. coli*, and the gram-positive *B. cereus*. **(A,C,E)** LPS challenge. **(B,D,F)** LTA challenge. **(A,B)** PR of total haemocyte. **(C,D)** PR of hyaline amoebocytes. **(E,F)** PR of granular cells. The PR of total haemocytes from control animals receiving MS once or twice (controls) was 17.9 ± 3.7% for *E. coli*, and 22.4 ± 1.8% for *B. cereus*; the PR of control hyaline cells was 1.5 ± 1.1 for *E. coli* and 3.5 ± 0.4 for *B. cereus*, while the PR of control granular cells was 16.5 ± 1.8 for *E. coli* and 19.0 ± 0.9 for *B. cereus*. The mean values ± SD of five animals for each treatment are reported.

The phagocytic rate (PR, percentage of phagocytosing cells within the phagocyte population) in control animals was between 17 and 24% towards both gram-negative (*E. coli*) and gram-positive bacteria (*B. cereus*) (see legend to Figure 3). Treatment of animals with LPS or with LTA induced a variable increase of the PR for both bacteria (dark grey columns, in the Figures 3A,B). After priming with either LPS or LTA, animals responded to an LPS challenge with increased PR towards both bacterial types (light grey columns, Figure 3A). Conversely, primed animals responded to an LTA challenge by down-regulating their PR towards both bacterial strains (light grey columns, Figure 3B). The memory-dependent PR variations in response to LPS and LTA was evident for both hyaline amoebocytes (Figures 3C,D) and granular cells (Figures 3E,F) for both bacteria in all homologous and heterologous priming/challenge combinations. To confirm the finding of challenge-dependent memory effects, we have run additional experiments using non-bacterial stimuli in the priming phase, i.e., the fungal β-glucan Zymosan and the virus-like dsRNA Poly(I:C), and LPS as challenge. Results reported in the Supplementary Figure 2A show that, independently of the priming agent, haemocytes respond to an LPS challenge with increased phagocytosis. This was true for total haemocytes (Supplementary Figure 2A) and for the two phagocytic subpopulations of hyaline and granular cells (Supplementary Figures 2B,C).

**Figure 4:**
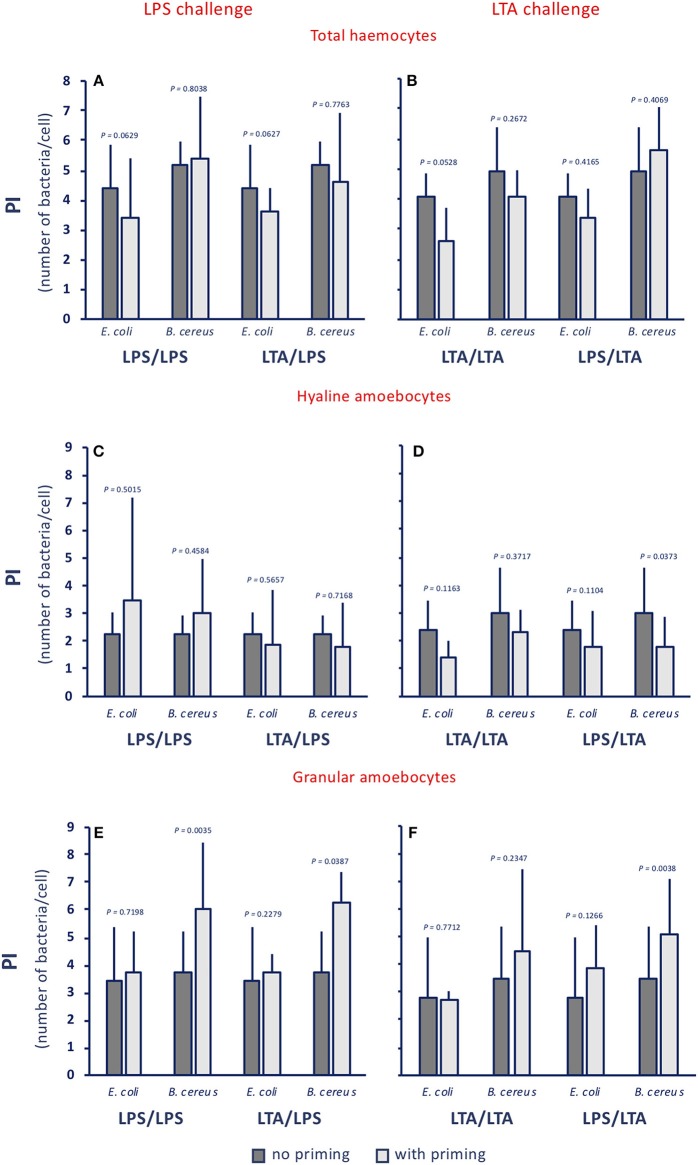
Innate memory-induced variations in the phagocytic index of haemocytes of *C. robusta* exposed *in vivo* to LPS or LTA. The phagocytic index (PI), i.e., the number of bacteria ingested by single phagocytes in haemolymph, was assessed 24 h after primary exposure to LPS or LTA (dark grey columns, no priming), and 24 h after homologous or heterologous challenge of primed animals (light grey columns, with priming). Phagocytosis of two bacterial strains was evaluated, the gram-negative *E. coli*, and the gram-positive *B. cereus*. **(A,C,E)** LPS challenge. **(B,D,F)** LTA challenge. **(A,B)** PI of total haemocyte. **(C,D)** PI of hyaline amoebocytes. **(E,F)** PI of granular cells. The PI of total haemocytes from control animals (treated with MS once or twice) was 1.6 ± 0.6 for *E. coli*, and 2.2 ± 0.4 for *B. cereus*; the PI of control hyaline cells was 0.9 ± 0.4 for *E. coli* and 1.5 ± 0.7 for *B. cereus*, while the PI of control granular cells was 3.0 ± 1.1 for *E. coli* and 3.4 ± 0.9 for *B. cereus*. The mean values ± SD of three animals for each treatment are reported.

When examining the phagocytic index (PI, i.e., the number of phagocytosed bacteria per cell), no memory effect is evident, as the number of ingested bacteria per cell does not change significantly (Figure 4A), despite a tendency of hyaline cells to phagocytose less bacteria, in particular upon LTA challenge (Figure 4B), and of granular cells to phagocytose more (Figure 4C). These results, showing the lack of memory-induced PI variations, were confirmed when using non-bacterial priming stimuli and LPS as challenge (Supplementary Figure 3).

Thus, although the memory effect does not depend on the type of priming stimulus, it seems to depend on the type of challenge, and is evident in a variation of the composition of the phagocyte population (different proportions of the more phagocytic granular cells and of the less phagocytic hyaline amoebocytes) that results in a variation of the PR. These memory effects on phagocytosis are non-specific at the level of the phagocytosed particles, since gram-negative and gram-positive bacteria are phagocytosed in the same way, and point towards enhanced phagocytosis upon LPS challenge, and decreased phagocytosis upon LTA challenge, implying that the two types of bacterial molecules trigger different defensive mechanisms.

### Innate Memory Affects the Expression of Immune-Related Genes

We assessed the expression of immune-related genes in *C. robusta* upon stimulation with the prototypical bacterial molecules LPS or LTA, and compared it to that of control animals and animals that were previously primed with either stimulus.

**Figure 5:**
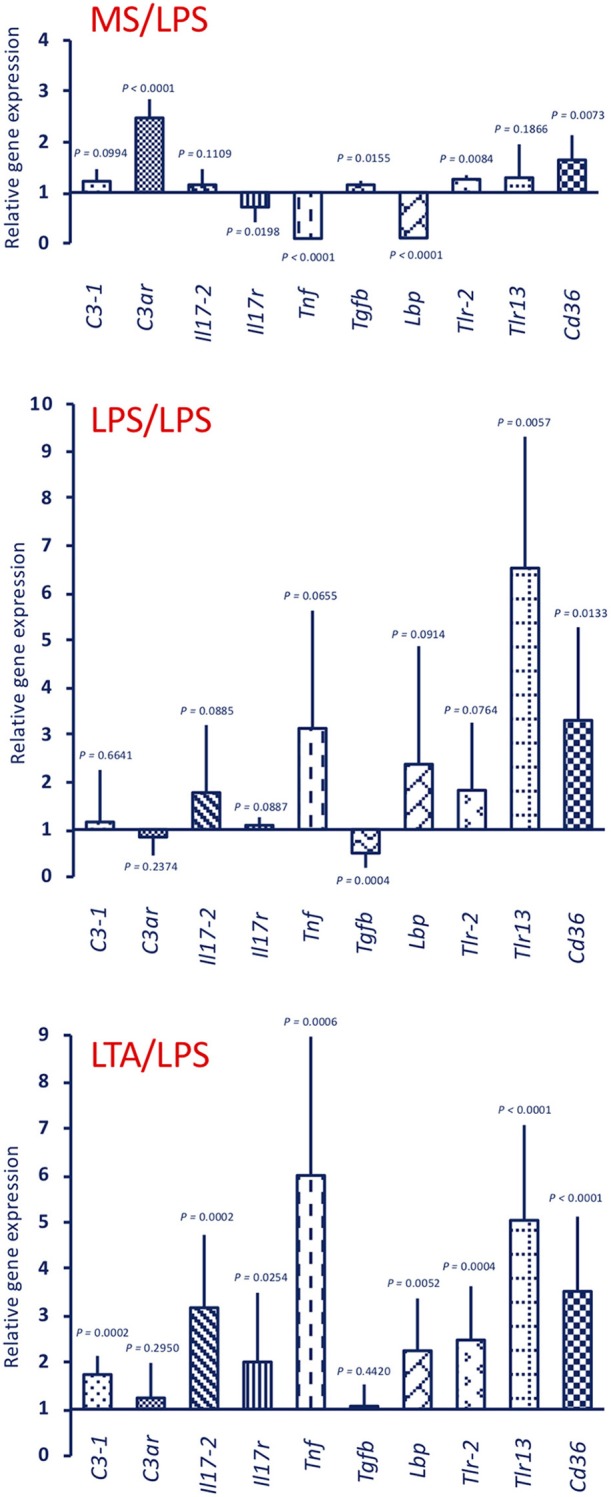
Innate memory-induced variations in the expression of immune-related genes in the pharynx of *C. robusta* challenged *in vivo* with the gram-negative stimulus LPS. LPS-induced expression in the pharynx of genes encoding the immune-related factors C3-1, C3aR, IL-17-2, IL-17R, TNFα, TGFβ, LBP, TLR-2, TLR13, and CD36 was measured in control MS-primed *C. robusta* animals (upper panel MS/LPS), and in animals that were previously primed with LPS (centre panel LPS/LPS) or LTA (lower panel LTA/LPS). Gene expression is reported relative to expression in control naïve animals. The mean expression values ± SD from three animals for each treatment are reported.

Response to an *in vivo* stimulation with 50 μg LPS of control animals (primed with MS alone) showed an up-regulation of *C3ar, Tlr-2*, and *Cd36* expression, a down-regulation of the *Il17r, Tnf*, and *Lbp* genes, and no variation in the expression of other genes (*C3-1, Il17-2, Tgfb, Tlr13*) (Figure 5, upper panel MS/LPS). LPS stimulation in animals that had been previously primed with LPS (Figure 5, centre panel LPS/LPS) or with LTA (Figure 5, lower panel LTA/LPS) caused a priming-induced re-shuffling of the gene expression profile. The priming-induced memory decreased the response to LPS in terms of expression of the *C3ar* gene, while it did increase expression of practically all other genes, except *Tgfb*, with notable increase of *Lbp, Tlr-2, Tlr13*, and *Cd36*.

**Figure 6:**
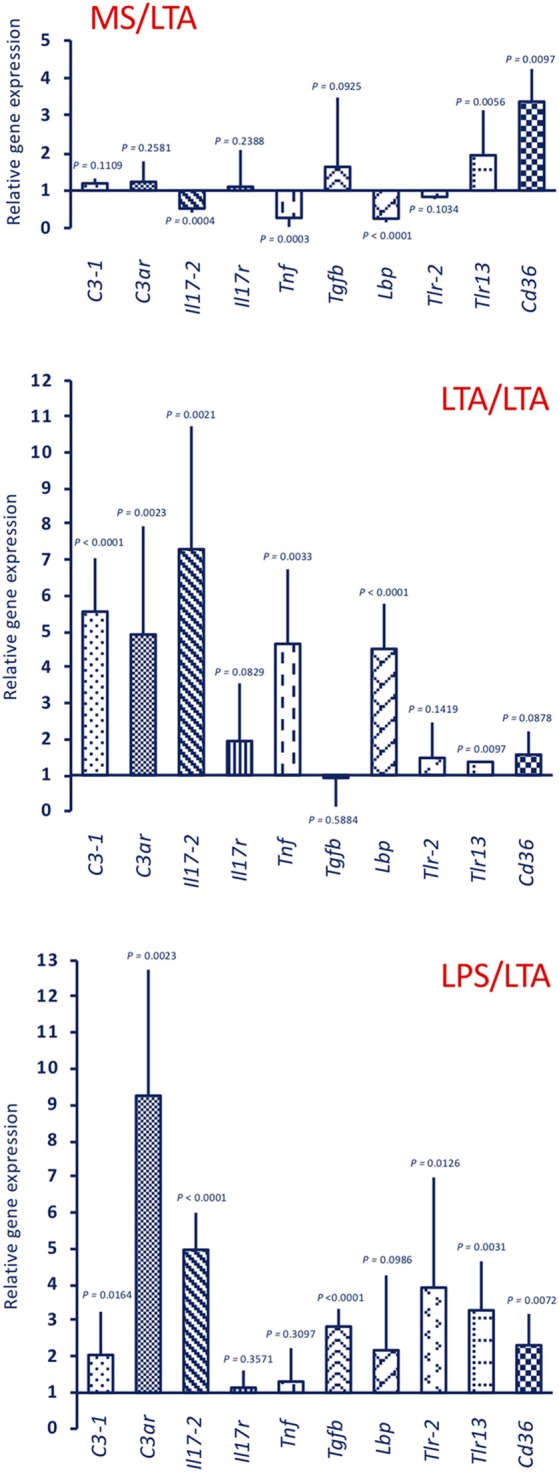
Innate memory-induced variations in the expression of immune-related genes in the pharynx of *C. robusta* challenged *in vivo* with the gram-positive stimulus LTA. LTA-induced expression in the pharynx of genes encoding the immune related factors C3-1, C3aR, IL-17-2, IL-17R, TNFα, TGFβ, LBP, TLR-2, TLR13, and CD36 was measured in MS-primed control *C. robusta* animals (upper panel MS/LTA) and in animals that were previously primed with LTA (centre panel LTA/LTA) or LPS (lower panel LPS/LTA). Gene expression is reported relative to expression in control naïve animals. The mean expression values ± SD from three animals for each treatment are reported.

Response to LTA induces a different gene expression profile (Figure 6, upper panel MS/LTA). *C3ar* gene expression was not changed, while there is a significant up-regulation of the cellular immunity-related genes *Tlr13* and *Cd36*. Similar to animals challenged with LPS, LTA-challenged animals had decreased *Tnf* and *Lbp* expression, whereas expression of other genes was substantially unaffected. In primed animals, the response to LTA showed a memory effect resulting in a significant increase in the expression of several genes (*C3-1, C3ar, Il17-2, Tnf*, and *Lbp*). A difference in the memory effect was evident depending on the priming stimulus, as LTA priming caused a decrease in the expression of *Tlr13* and *Cd36* upon LTA challenge (Figure 6, centre panel LTA/LTA), whereas LPS priming did not affect the LTA-induced expression of these genes, and significantly increased *Tgfb* expression (Figure 6, lower panel LPS/LTA).

**Figure 7:**
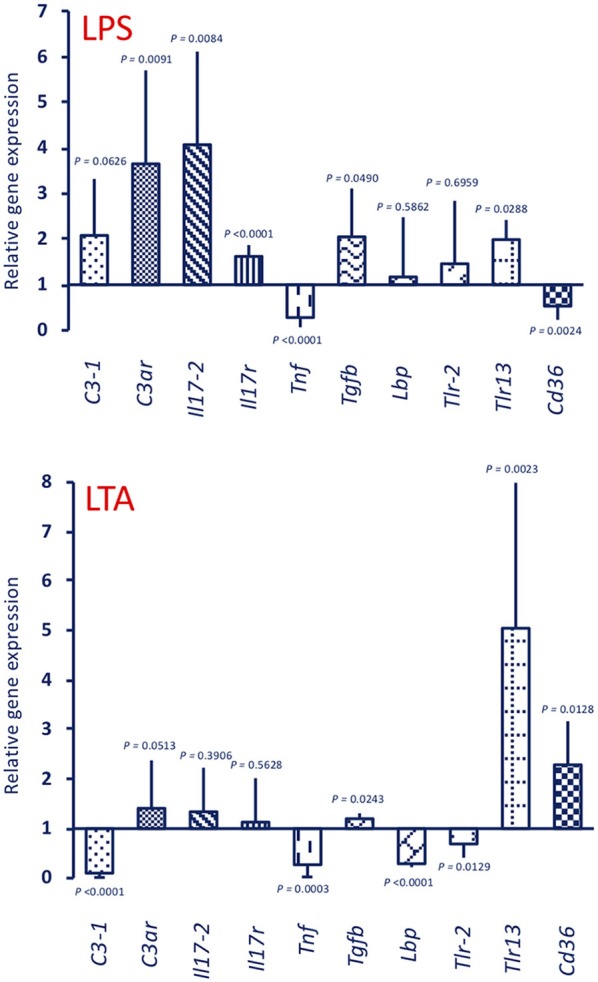
Expression of immune-related genes in the pharynx of *C. robusta* primed *in vivo* with bacterial stimuli. Expression in the pharynx of genes encoding the immune-related factors C3-1, C3aR, IL-17-2, IL-17R, TNFα, TGFβ, LBP, TLR-2, TLR13, and CD36 was measured in *C. robusta* animals 24 h after *in vivo* administration of 25 μg of LPS (upper panel LPS) or 25 μg of LTA (lower panel LTA). Gene expression is reported relative to expression in control naïve animals. The mean expression values ± SD from three animals are reported.

Stimulation with 25 μg LPS (the dose used for priming) of unprimed animals generated a gene expression profile (Figure 7, upper panel LPS) that is similar to that of the MS/LPS animals (Figure 5 upper panel MS/LPS), with significant up-regulation of humoral immune-related genes and down-regulation of the *Tnf* gene. Likewise, stimulation of unprimed animals with 25 μg LTA (the dose used for priming) (Figure 7, lower panel LTA) generated a gene expression profile similar to that of the MS/LTA animals (Figure 6, upper panel MS/LTA), with significant up-regulation of the cellular immunity-related genes *Tlr13* and *Cd36*, and down-regulation of the *Tnf* and *Lbp* genes.

A summary of the memory-dependent changes in gene expression, relative both to primary response and baseline expression, is reported in Supplementary Tables 1–3, which list the priming-specific (Supplementary Table 1), the challenge-specific (Supplementary Table 2), and the priming/challenge-specific variations (Supplementary Table 3). It is noteworthy that some specificity (i.e., changes peculiar of LPS *vs*. LTA priming) can be observed in priming-induced memory in response to any challenge (Supplementary Table 1), while a significant number of changes were dependent on the challenging stimulus, but independent of the type of priming (Supplementary Table 2). Eventually, a number of priming/challenge-specific changes were also evident (Supplementary Table 3). Overall, the up-regulation of *Tnf* and *Lbp* was the only change that occurred in all priming/challenge combinations.

## Discussion

This study provides the first formal evidence of innate immune memory in the ascidian *C. robusta*. An old excellent study, aimed at assessing the effect of age on immune responses, has already suggested that *C. robusta* could have immune memory (27). That study showed that the response to the injection of foreign erythrocytes shifted from phagocytosis to encapsulation upon subsequent stimulations (28). Although the authors did not consider their results as evidence of immune memory, we think that the maturation of a defensive response from a first type of reaction (phagocytosis) to an allegedly more efficient defensive mechanism (encapsulation) is suggestive of immune memory. After that first suggestion, the present study shows that *C. robusta* develops an innate immune memory that allows for a different kind of response upon repeated exposure to microbial agents. In this, we have the advantage of new knowledge and tools, such as the availability of the *C. robusta* genome, and the identification of a number of genes highly homologous to vertebrate immune-related genes that can allow us to describe immune-related pathways (20, 21, 24, 37).

The aim of our study is to describe immune memory in *C. robusta*, its level of specificity, and its possible underlying mechanisms. In this study we have used prototypical microbial agents as stimuli, due to the fact that *C. robusta* has not known pathogens. For the same reason, the endpoints for assessing immune memory establishment cannot include survival upon infection with pathogens. Thus, in the majority of our experiments we have used as stimuli two prototypical inflammatory bacterial components, LPS from gram-negative and LTA gram-positive bacteria, to mimic possible exposure to infectious agents, and we have examined memory establishment both at the functional level (phagocytosis) and at the level of immune-related gene expression.

By examining functional phagocytosis data, there are a number of issues that are worth underlining. First, there is a difference in the memory effects at the level of percentage of phagocyte subpopulations (granular *vs*. hyaline cells) and their Phagocytic Rate (PR) as opposed to their Phagocytic Index (PI). In fact, a memory effect is evident in the variation of percentage of phagocyte subpopulations and on their PR, whereas memory-induced variations were not generally evident on the PI. This strongly suggests that the memory effect on phagocytosis is largely at the level of changing the number of phagocytic cells (which may occur through mechanisms such as haematopoiesis, mitosis, differentiation or haematocytosis). This would exclude the mechanisms of epigenetic re-programming of specific cell functions, which are thought to be the main basis of several innate immune memory phenomena in vertebrates (50, 51). It is worth noting that both gram-negative and gram-positive stimuli increased the percentage of phagocytic haemocytes. Upon challenge with LPS, the number of granular amoebocytes (the most efficient phagocyte subpopulation) increased significantly, whereas upon LTA challenge granular amoebocytes decreased and hyaline cells increased. The memory-induced increase in granular cells in response to LPS was non-specific as it could be induced not only by priming with LPS, but also by LTA (Figure 2), Zymosan and Poly(I:C) priming (not shown). Thus, although the percentage of phagocytes is increased by treatment, its composition differs depending on the stimulus, shifting towards more efficient phagocytosis (i.e., more granular cells) upon LPS challenge. Indeed, the PR shows that upon LPS challenge phagocytosis is increased towards both gram-negative and gram-positive bacteria. Overall, the effect seems to be independent of the priming stimulus, while it depends on the type of challenge. This was confirmed by results obtained by priming animals with fungal or viral-like stimuli, showing that the increased phagocytosis induced by the LPS challenge occurred independently of the nature of the priming stimulus. This leads us to hypothesize that priming (with any inflammatory stimulus) induces a different capacity of the haematopoietic system (or resident haemocytes/phagocytes) to react to subsequent challenges. The type of subsequent reaction, however, fully depends on the type of challenge, with LPS inducing a preferential recruitment/differentiation of granular phagocytes, and LTA provoking a higher recruitment/differentiation of hyaline cells. We do further hypothesize that this outcome is instrumental to a more efficient protection from infections, meaning that phagocytosis with the involvement of granular cells is the major effector mechanism for tackling gram-negative infections (thereby justifying its memory-dependent increase induced by LPS challenge), whereas more efficient defence against gram-positive infections (as expected upon memory-driven responses to LTA) is associated with less phagocytosis, and the likely shifting to more efficient defensive mechanisms (such as complement-dependent bacteriolysis), possibly associated to hyaline cells.

The establishment of innate memory was tested also at the level of expression of immune-related genes. As already mentioned, most of the immune-related genes and pathways identified in *C. robusta* (as well as in many other invertebrates) are based on homology with vertebrate genes whose protein products have been functionally characterised. Indeed, with the exception of some complement components (20, 21), proteins encoded by these genes have not been identified in *C. robusta*, and therefore their real function is unknown. Thus, the immune-related gene expression profiles that we have generated in this study may provide a general idea of the memory effects in regulating immune reactivity, but the real impact will require a thorough functional validation with tools that are presently unavailable.

Based on the homology with vertebrate genes, we show that the primary response to LPS results in a minimal up-regulation of genes involved in cellular immune reactions (*Tlr-2, Tlr13, Cd36*), while in primed animals (with either LPS or LTA) LPS induces a highly increased expression of these genes. Priming with LPS also results in an up-regulation of the *Tnf* and *Lbp* gene upon challenge (with either LPS or LTA).

The primary response to LTA is very different from that triggered by LPS. There is no up-regulation, compared to basal expression, of genes for C3-1, C3aR, IL-17, and IL-17R (which are up-regulated by LPS), while there is up-regulation of *Tlr13* and *Cd36* (which are only marginally affected by LPS). In primed animals (with either LTA or LPS), the LTA response profile develops into a general activation, with the up-regulated expression of *C3-1, C3ar, Il17-2, Tnf*, and *Lbp* genes. Overall, it may be hypothesized that memory pushes the response to LTA towards enhanced complement-mediated and inflammatory effector reactions, compared to the primary response in which a main defensive role could be ascribed to cell-mediated mechanisms (TLR13, CD36).

While the gene expression profiles at challenge are expected to bring about improved protective responses, the differences between memory-induced effects in response to LPS and LTA suggest that this can be achieved by engaging different mechanisms. By comparing the response to LPS of control animals to that of animal previously primed with either LPS or LTA (Figure 5), it is evident that the memory response shifts from an essentially humoral (complement-dependent) response to an inflammatory and cellular reaction. Conversely, in the case of response to LTA (Figure 6), memory induces a shift towards humoral immunity There are however some points in common. For instance, expression of *Tnf* and *Lbp* genes appeared to be always up-regulated upon challenge, relative to the primary response.

**Figure 8:**
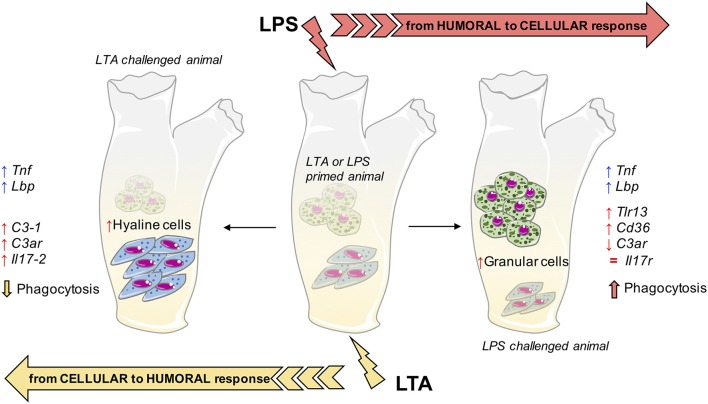
Innate memory mechanisms in *C. robusta*. Schematic representation of the putative mechanisms underlying innate memory in *C. robusta*, based on the results obtained in this study. Primed animals (with either LPS or LTA; centre), when challenged with LPS experience a general shift towards cellular response **(right)**, while challenge with LTA induces a general shift towards humoral response **(left)**. The shift is evaluated functionally (phagocytosis), in terms of relative number of granular vs. hyaline amoebocytes, and as increased (↑), decreased (↓), or normalised (=) expression of immune-related genes. Two genes, i.e., *Tnf* and *Lbp*, are always up-regulated in primed animals independently of the type of challenge.

It is of particular interest to compare the memory-induced immune-related gene expression profiles with the memory-induced functional defensive profile (phagocytosis). As already mentioned, the phagocytic response at challenge seems to mainly depend on the type of challenge rather than the type of priming, implying non-specific memory mechanisms. By examining the challenge-dependent memory profiles in gene expression (Supplementary Table 2), there are two genes whose expression correlates with phagocytosis. The complement receptor gene *C3ar* shows an inverse correlation, i.e., is down-regulated when phagocytosis is enhanced (combinations LPS/LPS and LTA/LPS) and up-regulated when phagocytosis is decreased (combinations LTA/LTA and LPS/LTA). A gene important in cell-mediated immunity, *Cd36*, shows a direct correlation with phagocytosis, i.e., is up-regulated with up-regulation of phagocytosis and *vice-versa*. Such correlations suggest that the *Cd36* gene product may be among the factors underlying the memory-dependent functional phagocytic response, and that phagocytosis is based on cellular immunity that is alternative to some humoral immunity factors, in particular complement. The summary in Figure 8 depicts the hypothesis of how memory could imply both distinct and common pathways to reach improved immune protection in *C. robusta*. Since infectious microorganisms in *C. robusta* have not been described yet, we will need additional information for proving this hypothesis in the future.

In conclusion, this study shows that exposure of *C. robusta* to microbial agents induces a reaction that primes animals for developing a different response (expectedly more protective) to subsequent challenges. This immune memory relies on the modulation of a number of different mechanisms, among which some common features can be identified. Indeed, memory responses imply an increased expression of the inflammation-related genes *Tnf* and *Lbp* upon subsequent challenges. On the other hand, memory-dependent responses to LPS are shifted towards increased recruitment of granular amoebocytes, more phagocytosis and enhanced expression of genes involved in cell-mediated immunity, while the memory response to LTA encompassed higher presence of hyaline cells, decreased phagocytosis and cellular immune responses in favour of increased complement-mediated and humoral immunity.

## Data Availability

The raw data supporting the conclusions of this manuscript will be made available by the authors, without undue reservation, to any qualified researcher.

## Author Contributions

DM and RM designed the study, performed the experimental study, evaluated the results, and contributed to writing the manuscript. GD contributed to performing the experimental study and to evaluating the results. PI designed the study, evaluated the results, and contributed to writing the manuscript. DB designed the study, evaluated the results, and wrote the manuscript.

### Conflict of Interest Statement

The authors declare that the research was conducted in the absence of any commercial or financial relationships that could be construed as a potential conflict of interest.

## Abbreviations

LPS: lipopolysaccharide gram-negative endotoxin
LTA: lipotheicoic acid from gram-positive bacteria
MS: marine solution
PAMP: pathogen-associated molecular pattern
PI: phagocytic index
PR: phagocytic rate
TLR: Toll-like receptor.
