# Supplementary material for: Assessing Immunological Memory in the Solitary Ascidian Ciona robusta
Source: Front Immunol. 2019 Aug 16;10:1977. doi: 10.3389/fimmu.2019.01977 (PMC6707023; doi:10.3389/fimmu.2019.01977)
Supplement: Supplementary file 1 [file Data_Sheet_1.PDF]

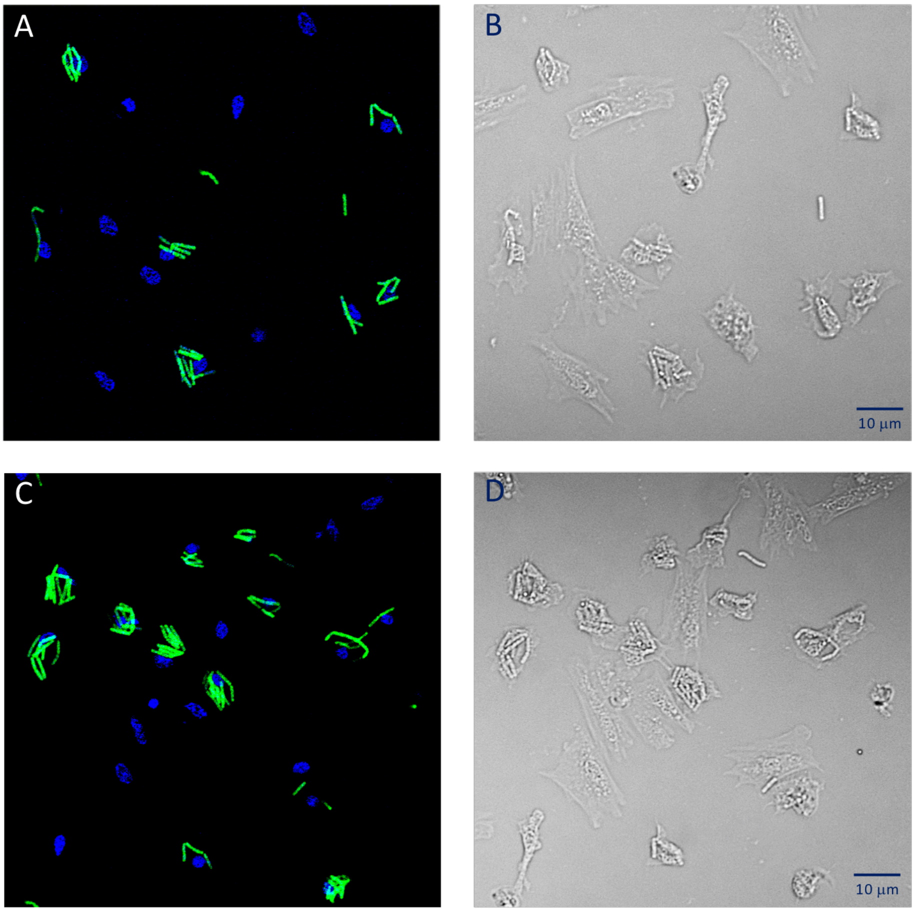

Supplementary Figure 1. Phagocytosis by *C. robusta* haemocytes.

Phagocytosis was performed *in vitro* by exposing *C. robusta* haemocytes to fluorescent *B. cereus* bacteria for 1 h at 18°C.

Panels A and B: haemocytes from control animals phagocytosing fluorescent bacteria.

Panels C and D: haemocytes from LPS-treated animals phagocytosing fluorescent bacteria.

Panels A and C: dark field, panels B and D: white field.

Haemocyte nuclei were labelled with the nucleic acid dye DAPI (blue colour), bacteria were labelled with FITC (green colour). Scale bar 10 µm.

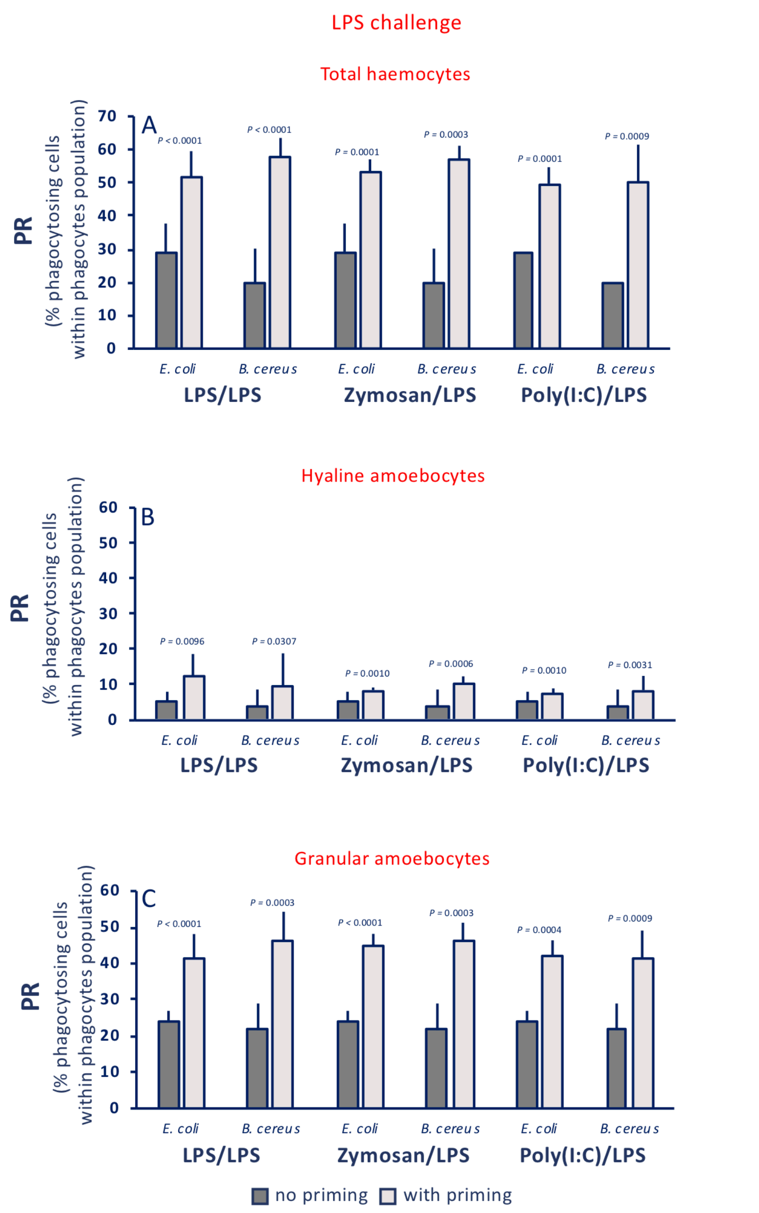

Supplementary Figure 2. Innate memory-induced variations in the phagocytic rate of *C. robusta* haemocytes challenged *in vivo* with LPS.

The phagocytic rate (PR), *i.e.*, the percentage of phagocytosing cells within the phagocyte population in haemolymph, was assessed 24 h after primary exposure to LPS, Zymosan or Poly(I:C) (dark grey columns, no priming), and after a 24-h LPS challenge of primed animals (light grey columns, with priming).

Panel A: PR of the total haemocyte population for two bacterial strains, the gram-negative *E. coli*, and the gram-positive *B. cereus*. The PR of control animals receiving MS once or twice (controls) was 17.9 ± 3.7% for *E. coli*, and 22.4 ± 1.8% for *B. cereus*. The mean values ± SD of 5 animals are reported.

Panel B: PR of hyaline cells, *i.e.*, one of the two phagocytic amoebocyte subpopulations. The PR of control hyaline cells (from animals receiving MS once or twice) was 1.5 ± 1.1 for *E. coli* and 3.5 ± 0.4 for *B. cereus*.

Panel C: PI of granular cells, *i.e.*, the other phagocytic amoebocyte subpopulation. The PR of granular cells (from animals receiving MS once or twice) was 16.5 ± 1.8 for *E. coli* and 19.0 ± 0.9 for *B. cereus*.

The mean values ± SD of 5 animals for each treatment are reported.

The data for the LPS/LPS combinations are the same reported in the Figure 3 and are repeated here for comparison.

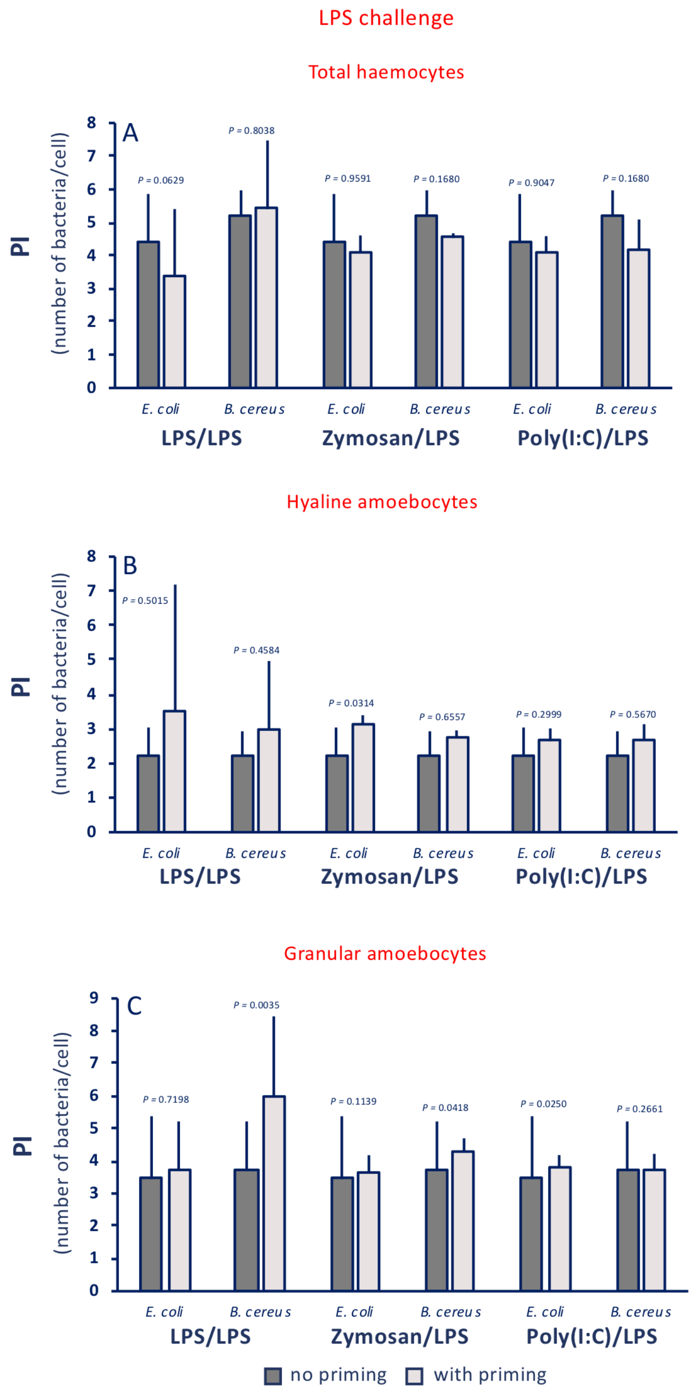

Supplementary Figure 3. Innate memory-induced variations in the phagocytic index of *C. robusta* haemocytes challenged *in vivo* with LPS.
The phagocytic index (PI), *i.e.*, the number of bacteria ingested by single phagocytes in haemolymph, was assessed 24 h after primary exposure to LPS, Zymosan or Poly(I:C) (dark grey columns, no priming), and after a 24-h LPS challenge of primed animals (light grey columns, with priming).
Panel A: PI of the total haemocyte population for two bacterial strains, the gram-negative *E. coli*, and the gram-positive *B. cereus*. The PI of haemocytes from control animals receiving MS once or twice (controls) was 17.9 ± 3.7% for *E. coli*, and 22.4 ± 1.8% for *B. cereus*. The mean values ± SD of 5 animals are reported.
Panel B: PI of hyaline cells, *i.e.*, one of the two phagocytic amoebocyte subpopulations. The PI of control hyaline cells 0.9 ± 0.4 for *E. coli* and 1.5 ± 0.7 for *B. cereus*.
Panel C: PI of granular cells, *i.e.*, the other phagocytic amoebocyte subpopulation. The PI of control granular cells was 3.0 ± 1.1 for *E. coli* and 3.4 ± 0.9 for *B. cereus*.
The mean values ± SD of 5 animals for each treatment are reported.
The data for the LPS/LPS combinations are the same reported in the Figure 4 and are repeated here for comparison.

Supplementary Table 1. Priming-dependent variations of gene expression levels in primed/challenged *C. robusta*

Gene	**Priming-dependent changes in the response to any challenge**		
	LPS priming-dependent changes	*LTA priming-dependent changes*	*Common priming changes*
C3-1	-	Up (above bkg)	-
C3ar	-	-	-
Il17-2	-	Up (above bkg)	-
Il17r	-	-	-
Tnf	-	-	Up (above bkg or normalised)
Tgfb	-	-	-
Lbp	-	-	Up (above bkg)
Tlr-2	-	-	-
Tlr13	-	-	-
Cd36	-	-	-

Supplementary Table 2. Challenge-dependent variations of gene expression levels in primed/challenged *C. robusta*

Gene	**Challenge-dependent changes in the response to any priming**		
	LPS challenge-dependent changes	*LTA challenge-dependent changes*	*Common challenge changes*
C3-1	-	Up (above bkg)	-
C3ar	Down (below bkg)	Up (above bkg)	-
Il17-2	-	Up (above bkg)	-
Il17r	Up (normalised)	-	-
Tnf	-	-	Up (above bkg or normalised)
Tgfb	-	-	-
Lbp	-	-	Up (above bkg or normalised)
Tlr-2	-	-	-
Tlr13	Up (above bkg)	-	-
Cd36	Up (above bkg)	-	-

Supplementary Table 3. Priming/challenge-specific variations of gene expression levels in primed/challenged *C. robusta*

Gene	**Priming-dependent changes in the response to individual challenges**			
	LPS priming/LPS challenge-dependent changes	*LTA priming/LTA challenge-dependent changes*	*LPS priming/LTA challenge-dependent changes*	*LTA priming/LPS challenge-dependent changes*
C3-1	none	-	-	-
C3ar	-	-	-	-
Il17-2	none	-	-	-
Il17r	-	-	-	-
Tnf	-	-	-	-
Tgfb	Down (below bkg)	-	Up (above bkg)	-
Lbp	-	-	-	-
Tlr-2	-	none	-	-
Tlr13	-	none	none	-
Cd36	-	Down (normalised)	-	-
